# Uptake and user characteristics of MyChart within a Canadian community hospital with a diverse patient population: A comparative study

**DOI:** 10.1371/journal.pdig.0000852

**Published:** 2025-05-12

**Authors:** Shelley Vanderhout, Shipra Taneja, Kamini Kalia, Terence Tang, Walter P. Wodchis

**Affiliations:** 1 Institute for Better Health, Trillium Health Partners, Mississauga, Ontario, Canada; 2 Institute of Health Policy, Management and Evaluation, University of Toronto, Toronto, Ontario, Canada,; 3 Trillium Health Partners, Mississauga, Ontario, Canada; Iran University of Medical Sciences, IRAN, ISLAMIC REPUBLIC OF

## Abstract

Patient portals offer a convenient way to access health information and increase patient participation in healthcare. To promote broad accessibility and impact of portals, it is essential to understand uptake patterns across patient populations. This study described the characteristics of patient users of a portal called MyChart and compared them to non-users at a large community hospital. We descriptively analyzed (frequency, counts) patient health records to characterize MyChart users and their usage patterns during the first year of its launch from September 11, 2023, to September 112024. We summarized user demographics along with information about how they activated accounts, accessed MyChart, and utilized its features. Using chi-square and t-tests, we compared MyChart user demographics to non-users who visited the hospital in the same time period. A total of 61,306 patients activated MyChart during the first year it was available. On average, MyChart users were 53 years old, 62% female, 64% predicted to have White ethnicity, and preferred to receive healthcare in English (88%). MyChart users tended to be regular healthcare users, with an average of five annual visits prior to creating an account and logged onto the portal on average five times a month. MyChart users were slightly younger than non-users (an average age of 53.5 vs. 56.9 years) and visited the hospital more often (an average of 5.7 vs. 3.1 annual visits). Many patients activated MyChart during the first year of launch, and users closely resembled the broader patient population. To enhance adoption and potential benefits of patient portals, targeted interventions such as accessible educational information tailored to diverse patient groups (e.g., older adults, different ethnicities) could increase their usage.

## Author summary

Patient portals offer a convenient way to access health information and increase patient participation in healthcare. However, their effectiveness can vary depending on factors such as implementation, misaligned expectations, limited digital and health literacy and privacy and security concerns. This study contributes to the existing literature by providing evidence of patient portal adoption and usage directly following its implementation in a large community hospital that serves a highly diverse patient population. It identifies specific user groups, such as older adults, who may require additional support to effectively utilize the portal, emphasizing the importance of caregiver involvement and a staged implementation approach. This study can guide future research by informing the development of targeted interventions that focus on specific user groups (e.g., older adults) or challenges (e.g., digital literacy) to improve patient portal engagement. Additionally, our findings inform healthcare practice and policy by highlighting the need for equitable access and usage of patient portals for all individuals, ultimately improving the quality and efficiency of healthcare delivery.

## Introduction

As healthcare systems increasingly adopt electronic health records, patient portals have emerged as an essential tool for patient engagement and care coordination [1,2]. These digital tools offer patients a secure and convenient way to access their personal health information by offering features such as viewing medical notes and test results, booking appointments, and completing questionnaires between visits [3]. These features can empower patients to be more active in managing their care and can lead to improved healthcare knowledge [4,5], medication adherence [4], enhanced patient-provider communication [5], and greater overall satisfaction with care [6]. Health systems can also benefit from patient portals by streamlining requests for information, allowing patients to update personal information and manage appointment schedules, and reducing avoidable patient calls and visits [7–9].

Despite the growing patient popularity of digital health tools, with over 39% of Canadians already using them and 81% expressing future interest, implementing patient portals remains a challenge for many healthcare systems [2,10,11]. Successful portal implementation requires a complex synergy of significant financial investment, leadership advocacy, healthcare provider buy-in, and patient trust and understanding of their purpose [12,13]. To drive the greatest return on these investments, sophisticated and long-term strategies are needed to ensure providers and patients are aware that a portal is available, learn how to sign up and use its numerous features, and understand how to incorporate it into care routines. Particularly in settings with diverse populations, patients may experience barriers to accessing and using portals due to factors such as limited digital and health literacy, language barriers, and limited support for learning to navigate them [12,14]. These challenges, including lack of access to resources like computers and reliable internet, past negative experiences with technology, and concerns surrounding the comprehension of complex health information and data security, can limit the potential benefits patient portals offer and may further exacerbate health disparities experienced equity-deserving groups [15]. Determining how many and which patients are using patient portals can help guide strategy refinement to ensure that certain patient populations are not inadvertently excluded from using and benefiting from them and guide the development of targeted interventions to help improve portal accessibility and uptake.

This study aimed to explore patient uptake and use of a patient portal called MyChart within the first year of its launch (from September 2023–2024) at a large community hospital. Specifically, we sought to:

1. Describe how many patients activated MyChart, what their characteristics were, and how they used MyChart.
2. Compare MyChart users’ characteristics to non-users.

## Methods

### Setting

This analysis was conducted at Trillium Health Partners (THP), a large community hospital with three locations in Mississauga, Canada. THP serves a diverse community, including many newcomers (51%) and visible minorities (62%) [16]. In the 2023–2024 fiscal year, the hospital recorded over 1.7 million patient visits (inpatient, outpatient and emergency visits) [16]. THP implemented Epic, a health information system, in October 2020 [17], and its patient portal, MyChart, was launched in September 2023 [3]. MyChart was available in English to all patients aged 12 years and older, and was advertised in English hospital waiting rooms and corridors, social media, and word of mouth. Parents or caregivers could create accounts for children under 12 years old as designated proxy users. Patients could activate a MyChart account through several methods, including receiving a link through a reminder email, finding a code on their after-visit summary, during an in-person encounter, or with assistance from staff. Patients could receive support for technical difficulties via email or view instructional videos online. THP patients had incremental access to various MyChart features over the first year of its roll-out (see S1 Fig), starting in September 2023 with viewing after-visit and discharge summaries, outpatient test results, and appointment schedules, electronically checking-in for appointments (“e-Check In”), and viewing or updating personal information, medications, allergies, and vaccinations. A few months after launch, patients could then modify appointment schedules, launch video visits, and renew prescriptions. A messaging feature between patients and providers was not activated at THP. This staged approach was taken to accommodate workforce capacity and dialogue with healthcare providers that indicated low readiness for many features to be introduced simultaneously, particularly messaging.

### Patient and public involvement

Prior to its launch, a patient partner group of 14 individuals helped to inform the MyChart implementation approach at THP. This group helped to inform communication strategies for informing patients about MyChart’s availability and features, decision making around which MyChart features to provide or limit access to, and guidance material for learning to use MyChart such as instructional videos. This group was offered early access to MyChart accounts to pilot test the user experience and provide feedback before the portal was made available to all THP patients.

### Data collection and analysis

We conducted a descriptive analysis of patient health records stored in Epic (hospital records) to describe characteristics of patients who activated MyChart and their usage patterns, as well as non-users of MyChart. All living patients who had an admission or outpatient (in-person or telephone) or emergency department visit from September 12, 2023, to September 11, 2024 were included in this analysis.We collect specific details, such as whether they had a MyChart account at THP, either created by themselves or a proxy (e.g., parent or caregiver), or those without an account (MyChart non-users). For MyChart users, we collected data on how patients activated MyChart accounts, whether they had a proxy linked, their last clinical encounter area and mode (virtual vs. in-person), how they accessed MyChart (mobile app vs. web browser), features they used such as video visits or e-Check In, which allows patients to provide relevant information using MyChart prior to an upcoming outpatient clinic visit. For all patients, we gathered patient first and last name, age, sex, preferred language, and visit frequency at THP in the last year. Patients’ racial identities were predicted using a probabilistic name-to-race prediction algorithm applied in Python. This algorithm, derived from United States census data, analyzed first and last names to generate race predictions by calculating the probability of a patient belonging to each race represented in the database, based on the frequency of their name within those groups. The race with the highest probability score was then selected.

[18,19] All data were descriptively summarized one year following MyChart implementation at THP. Missing data was reported, and no other statistical adjustments made to address missing values in the analysis. Postal codes were used to measure socio-economic measures of material deprivation (representing education, low income, unemployment, lone-parent families and dwellings in need of major repair) and ethnic concentration (representing immigrants within the past 5 years and visible minorities) based on patient neighbourhood, and categorized as quintiles, using the Ontario Marginalization Index (ON-MARG) [20]. Using electronic health record data, we extracted the demographics of MyChart users to non-users during the study period (name, age, sex, postal code, and preferred language), and then conducted Chi-square and t-tests for differences between users and non-users across age, sex, preferred language, frequency of hospital encounters, predicted race, neighbourhood material resources, and neighbourhood proportion of racialized and newcomer populations. Statistical significance was set using an alpha of 0.05. Microsoft Excel 2016 and SAS version 9.4 was used for statistical analysis and data management.

### Ethics

This study was reviewed by the THP Research Ethics Board and classified as quality improvement based on our primary purpose which was to explore and improve the delivery of healthcare services following their implementation.

## Results

### Participant characteristics and activation methods

In the first year of MyChart’s implementation from September 2023–2024, 61,306 patients activated MyChart accounts, representing 31.8% of all patients who visited the hospital during that time. User characteristics are described in Table 1, which we compared to non-users (n = 133,479) who visited the hospital for admission, outpatient appointments or emergency room visits during that same time period. On average, MyChart users were 53 years old, 62% were female, 64% were predicted to have White racial identity, and their preferred language was predominantly (88%) English. Fifty-one percent of MyChart-active patients were from the two lowest quintile areas for material resources, which are regions where residents may face challenges in securing basic material needs relating to housing, food, clothing and education. The ON-MARG Index also revealed that 76% of MyChart-active patients lived in the highest two quintile areas for relative proportions of racialized and newcomer groups. Very few (n = 232, 0.4%) MyChart accounts included proxy users. Statistical testing between users and non-users showed that there was evidence of differences between the groups across all demographics, even when proportions of users and non-users were similar. However, there were substantive differences in age and healthcare utilization: compared to non-users, MyChart users were slightly younger (53.5 vs. 56.9 years), and had more hospital visits in the past year (5.7 vs. 3.1 visits).

**Table 1:** Comparison of MyChart-active patients’ demographics to the patients who had visited our hospital at least once while MyChart was available.

Characteristic	MyChart users n = 61,306	Non-users n = 133,479	p-value [df, t-value or X2]	Patient population n = 194,785
Age, mean (SD)	53.5 ± 18.3	56.9 ± 21.3	<0.001 [136970, -35.39]	55.8 ± 20.5
Age range, n (%)			< 0.001 [5, 6364]	
0-17	1421 (2.3%)	9085 (6.8%)		10506 (5.4%)
18-40	15663 (25.6%)	19022 (14.3%)		34685(17.8%)
41-60	19455 (31.7%)	37123 (27.8%)		56578 (29.1%)
61-80	21354 (34.8%)	53957 (40.4%)		75311 (38.7%)
81-100	3403 (5.6%)	14255 (10.6%)		17658 (9.1%)
101+	10 (0.02%)	37 (0.03%)		47 (0.02%)
Sex, n (%)			< 0.001 [2, 216]	
Male	23105 (37.7%)	54986 (41.2%)		78091(40.1%)
Female	38183 (62.3%)	78440 (58.7%)		116623 (59.9%)
Other	18 (0.03%)	51 (0.04%)		71 (0.04%)
Preferred language (top 10, n (%))			< 0.001 [9, 390]	
English	54465 (90.3%)	115863 (88.6%)		170328 (89.2%)
Arabic	196 (0.3%)	820 (0.6%)		1016 (0.5%)
Punjabi	177 (0.3%)	854 (0.1%)		1031 (0.5%)
Chinese (Mandarin)	154 (0.3%)	638 (0.5%)		792 (0.4%)
Spanish	150 (0.3%)	525 (0.4%)		675 (0.4%)
Polish	95 (0.2%)	454 (0.4%)		549 (0.3%)
Urdu	153 (0.3%)	376 (0.3%)		529 (0.3%)
Portuguese	98 (0.2%)	404 (0.3%)		502 (0.3%)
Vietnamese	95 (0.2%)	362 (0.3%)		457 (0.2%)
Ukrainian	99 (0.2%)	331 (0.3%)		430 (0.2%)
Predicted racial identity (top 5, n (%))^*^			<0.001 [4,115]	
White	39171 (63.9%)	86330 (64.7%)		125501 (64.4%)
Asian Pacific Islander	15995 (26.1%)	32295 (24.2%)		48290 (24.8%)
Hispanic	4022 (6.6%)	9614 (7.2%)		13636 (7.0%)
Black	2038 (3.3%)	5022 (3.8%)		7060 (3.6%)
American Indian and Alaska Native	59 (0.1%)	171 (0.1%)		230 (0.1%)
Frequency of hospital encounters in the past year			<0.001 [58485, 39]	
Mean (SD)	5.7 ± 12.4	3.1 ± 8.8		3.7 ± 9.8
Median	2	1		2
Neighbourhood material resources, quintile (%)			<0.001 [5, 595]	
1 = lowest neighbourhood material resources	13357 (21.8%)	25975 (19.5%)		39332(20.2%)
2	17595 (28.7%)	35107 (26.3%)		52702 (27.1%)
3	15197 (24.8%)	33244 (24.9%)		48441 (24.9%)
4	9200 (15.0%)	22303 (16.7%)		31503 (16.2%)
5 = highest neighbourhood material resources	5418 (8.9%)	15601 (11.7%)		21019 (10.8%)
Unmatched	540 (0.9%)	1248 (0.9%)		1788 (0.9%)
Racialized and newcomer populations, quintile (%)			<0.001 [5,49]	
1 = lowest neighbourhood proportion of racialized and newcomer populations	1213 (1.7%)	2253 (1.9%)		3466 (1.8%)
2	3875 (5.8%)	7716 (6.3%)		11591 (5.9%)
3	9179 (14.7%)	19657 (14.9%)		28836 (14.8%)
4	16022 (26.4%)	35232 (26.1%)		51254 (26.3%)
5 = highest neighbourhood proportion of racialized and newcomer populations	30478 (50.5%)	67372 (49.7%)		97850 (50.2%)
Unmatched	540 (0.9%)	1248 (0.9%)		1788 (0.9%)

*Approximate racial identity calculated by last name using US census data[18,19]

**Fig 1:**
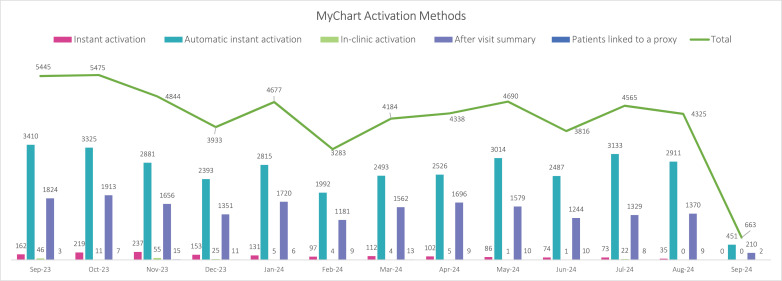
MyChart activation methods* by month from September 12, 2023, to September 11, 2024.

Legend: *Instant activation = Staff can send patients or proxies a text message or email with a link that allows them to activate MyChart; Automatic instant activation = for upcoming visits, patients are automatically sent MyChart activation links via email appointment reminders; In-clinic activation = patients use a provider’s computer to create a MyChart account during a visit; After visit summary = printed visit summaries include a unique code that patients can enter from their device to activate MyChart; Patients linked to a proxy = a parent or caregiver created a MyChart account on another person’s behalf.

Most patients activated MyChart accounts using links they received in email reminders prior to an upcoming health care encounter (Fig 1). MyChart users had an average of five health care encounters in the year prior to activating MyChart, and most often, they last visited diagnostic imaging (n = 29,844; 49%), followed by oncology (n = 4,704; 8%) and outpatient and renal medicine (n = 4,009; 7%) before MyChart activation.

### MyChart usage

**Fig 2:**
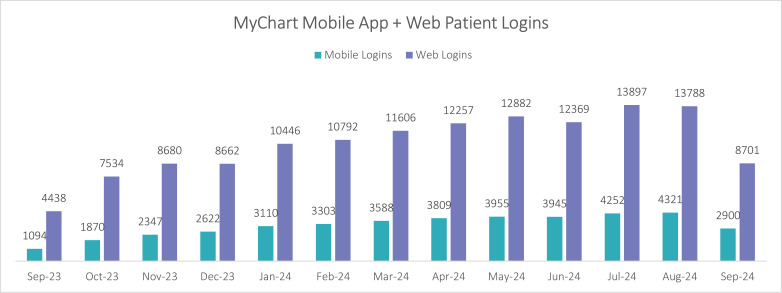
Monthly total MyChart logins by mobile app and web browser.

Patients logged in to MyChart an average of 5.4 times per month, and 24% of these logins were via the MyChart mobile app from a smartphone or tablet (Fig 2); the remainder were by web browser. Patients actively engaged with MyChart, conducting an average of 1510 video visits per month. e-Check In usage gained popularity over time; at its highest usage, this feature was used in 10.5% (n = 11,020/104,465) of all outpatient clinic appointments in the final month of our analysis. While using the e-Check In feature, patients updated their medications (n = 8,956), allergies (n = 5,957), personal information (n = 6,038), patient contacts (n = 5,957), and completed questionnaires (n = 9,271).

## Discussion

In its first year of implementation, over one third of patients who received care at a large community hospital in Mississauga, Canada activated MyChart accounts. On average, they were predominantly female, White racial identity, English-speaking, and middle-aged, which is consistent with other patient portal users described in the literature [21]. MyChart users tended to be regular healthcare users, with an average of five hospital visits in the year prior to creating an account and they logged on to the portal an average of five times per month. Compared to non-users, they were slightly younger and visited the hospital more often. Features such as virtual visits and e-Check In (available since September 2023) saw a significant growth in use over time, suggesting that patients gained comfort with the portal and continued to engage with it beyond activating accounts.

When we compared MyChart users to non-users, many of the demographics across the two groups were similar. Though statistical testing showed evidence of differences between users and non-users, we attribute this to a large sample size. Therefore, we focused on observable, substantive differences in our interpretation: MyChart users tended to be younger and visited the hospital more often than non-users. These findings align with some other literature showing that younger populations are more technologically literate and likely to use patient portals [22,23]; however, other studies have shown that MyChart users tend to be older [24,25] which may be correlated with higher healthcare usage that accompanies ageing. The literature about healthcare utilization and patient portal use is also mixed but may represent effects of different stages of portal use, where people with more healthcare encounters have more opportunities to learn about portals and receive invitations to activate accounts, and may have a higher desire or need for the information presented in portals [26,27]. Over time, though, studies show that users are less likely to visit emergency departments and may avoid unnecessary visits that portal features such as messaging may replace [28–30]. Notably, our findings also highlight potential disparities in access and engagement for older adults, less frequent healthcare users and non-English speaking population. This aligns with the broader concern about the digital divide, where factors such as age, language proficiency, and health literacy can create barriers to accessing and utilizing digital health tools [15].

Consistent with findings from other studies [31,32], we found that very few patients with MyChart added proxy users to their accounts, suggesting that awareness of this feature may be low or may have reflected local challenges with verifying and approving proxy users, or that caregivers activated MyChart accounts on others’ behalf and circumventing the proxy feature. The low number of children (under 12 years of age) with MyChart in our hospital likely reflected difficulties in adding proxy users to children’s accounts and low awareness among healthcare providers about how to activate them through a parent or caregiver. Nonetheless, children aged 13 years and over are often able to navigate digital health tools and manage health information [33], so efforts to enable MyChart use among this population could be beneficial for children at our hospital. While it is beneficial for caregivers to have portal access, sharing passwords presents a risk of inappropriate or unauthorized access to personal health information, especially among vulnerable populations such as children, seniors, or those with limited English proficiency [31,32]. Health systems should consider sharing additional communication and instructions about adding proxies to reduce this risk and help protect information privacy.

We identified considerable interest in MyChart among patients, and this may have been driven by existing familiarity with similar portals from other health systems, advances in patient digital literacy and access to devices and internet, or a growing culture around patient access to personal health records. Our staged approach to making MyChart features available was unique; many hospitals enable all features at once. This method was chosen to balance the impact of learning to use MyChart on front-line staff and provide a buffer to correct any technical or user experience issues that arose as the portal was implemented. As our hospital continues to explore MyChart uptake and use, the flexibility of this approach provides the opportunity to respond to patient demographics, preferences, and priorities. This may include disseminating educational information to staff, offering MyChart in additional languages, and actively engaging with members of diverse communities to ensure they have the resources and support to utilize MyChart if desired. However, MyChart usage may falter over time if patients perceive its functionalities to be too limited, especially when compared to what is offered at other hospitals.

### Limitations

There were limitations to this study. Though we felt it was important to predict patient race given the diversity of our population, this approach is based on a name-based algorithm derived from United States census data and may not have been entirely accurate in a Canadian context, as it is affected by factors such as names shared across multiple groups, variations in name spelling, and differences between US and Canadian demographics. We also sought to identify whether language barriers existed in portal access, but relied on EMR data about patients’ preferred language, which may not be accurate. Our hospital is situated in a community where over half of the population speaks English as a second language [16], so we estimate that our preferred language data may need updating and will explore this potential barrier in future research. Additionally, MyChart advertisements and enrollment support were available exclusively in English, potentially hindering uptake among patients with limited English proficiency, or those who speak English as a second language but prefer healthcare services in another language. Given the relatively short time period of this study and lack of data available, it is difficult to describe ongoing MyChart use and distinguish between patients who activated accounts once and those who use it regularly. Future studies that incorporate long-term data will help understand usage patterns and explore how to improve patient portal features and accessibility.

Our study offers actionable recommendations for healthcare systems looking to enhance MyChart implementation and utilization. We found that while MyChart users predominantly spoke English, a subset non-users preferred other languages. To address this equity challenge, healthcare systems should expand MyChart's multilingual interface and content to ensure accessibility for diverse populations. Targeted outreach in different languages, using advertisements and educational materials can help raise awareness and encourage adoption among non-English speakers. Additionally, digital and health literacy programs designed for older adults and non-English speakers can teach practical skills and overcome language and health literacy barriers. Our findings also suggest that many patients did not sign up for MyChart, which could be due to limited awareness or lack of understanding of its potential benefits [12]. Community engagement initiatives, like workshops in community centres, and hospital-led educational campaigns, such as in-clinic demonstrations, patient ambassadors, or social media marketing could help promote adoption, use, and trust in patient portals. Lastly, our findings suggest that caregivers may be unaware of proxy use on MyChart. Creating targeted educational materials such as short videos or how-to guides, and interventions, such as a workshops or proactive system prompts within MyChart can improve caregiver engagement and patient care.

## Conclusion

In the first year of its launch, we identified considerable patient interest in activating MyChart, and that typical MyChart users were both similar to those described in the literature and our broader patient population. However, there is still room to engage specific groups such as youth, those who speak languages other than English, and older adults to support MyChart use if there is interest, and ensure caregivers are accessing accounts as proxy users to promote privacy. We undertook a unique staged approach to offering various MyChart features, balancing capacity of the health system workforce and allowing for dialogue between patients, healthcare providers, and health system operators about what worked well and opportunities for improvement. Analysis of MyChart use over time will continuously guide how our health system engages patients and caregivers in care using this portal. Future research should include longitudinal studies to understand sustained patient portal engagement and the impact of targeted interventions to increase their uptake and use, such as simplified proxy access, multilingual resources (including videos, how to guides, and workshops), and interfaces available in multiple languages.

## Supporting information

S1 Fig MyChart feature roll-out. (DOCX)
