# Supplementary figures and images for: Uptake and user characteristics of MyChart within a Canadian community hospital with a diverse patient population: A comparative study
Source: PLOS Digit Health. 2025 May 12;4(5):e0000852. doi: 10.1371/journal.pdig.0000852 (PMC12068696; doi:10.1371/journal.pdig.0000852)

S1 Fig. MyChart feature roll-out.


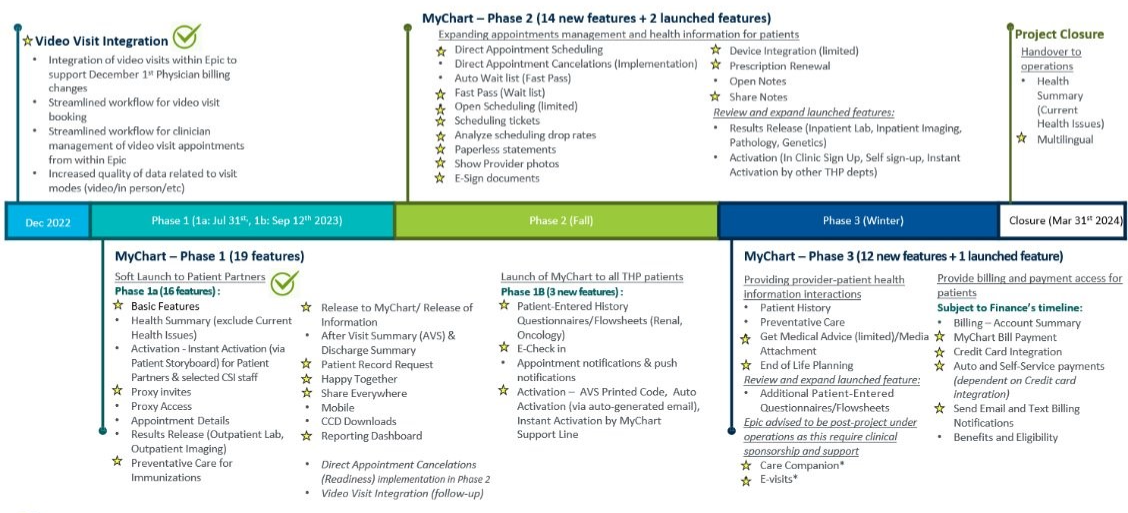

Supplement: S1 Fig — (DOCX) [file pdig.0000852.s001.docx]
